# Prevalence and Risk Factors of Hypertension in Hargeisa, Somaliland: A Hospital-Based Cross-Sectional Study

**DOI:** 10.3390/diseases11020062

**Published:** 2023-04-11

**Authors:** Faisal Nooh, Mohamed I. Ali, Afona Chernet, Nicole Probst-Hensch, Jürg Utzinger

**Affiliations:** 1 Swiss Tropical and Public Health Institute, Kreuzstrasse 2, CH-4123 Allschwil, Switzerland; 2 University of Basel, CH-4003 Basel, Switzerland; 3 College of Medicine & Health Sciences, University of Hargeisa, Hargeisa 25263, Somaliland; 4 College of Medicine & Health Sciences, Jigjiga University, Jigjiga 1020, Ethiopia

**Keywords:** cardiovascular diseases, cross-sectional study, hypertension, non-communicable diseases, prevalence, risk factors, Somaliland

## Abstract

Hypertension is the leading risk factor for cardiovascular diseases and represents a major public health challenge worldwide. There is a paucity of information regarding the hypertension status of adults in Somaliland. We aimed to assess the magnitude of, and factors associated with, hypertension among adult patients seeking care at Hargeisa group hospital in Hargeisa city, Somaliland. We conducted a health facility-based cross-sectional study enrolling adult outpatients. We used the World Health Organization (WHO) STEPwise surveillance approach to obtain patient information. A total of 319 participants (54.2% males; mean age 40.4 ± 14.0 years) had complete data records. The prevalence of hypertension was 22.6% (95% confidence interval; 18.2–27.6%). The prevalence of hypertension increased with age and was higher in males (24.9%) than in females (19.9%). Age, cholesterolaemia and obesity were significantly associated with hypertension. Separate analyses for females and males revealed that obesity was significantly associated with hypertension in females but not in males. On the contrary, cholesterolaemia was significantly associated with hypertension in males but not in females. We found a high prevalence of hypertension and multiple risk factors for non-communicable diseases (NCDs) in outpatients seeking care in Hargeisa. Our findings emphasise the need for enhanced focus on the prevention and management of NCDs in Somaliland.

## 1. Introduction

Globally, hypertension is the leading preventable risk factor for cardiovascular diseases (CVDs) [1,2]. It is currently estimated that 11 million deaths are attributable to hypertension every year, which accounts for 19.2% of the annual death toll [3]. In 2019, approximately 1.28 billion people had hypertension [4]. Over one billion hypertensive people were concentrated in low- and middle-income countries (LMICs) [4]. The Global Burden of Disease Study (GBD) 2020 reported a 5% increase in the prevalence of high blood pressure from 2010 to 2019 [2]. This increase in the prevalence of hypertension is primarily attributable to population growth and demographic ageing, coupled with changes in behavioural risk factors, such as alcohol consumption, smoking behaviour, physical inactivity, exposure to unhealthy diet and obesity [5]. Obesity and overweight increased by 18% in just one decade from 2010 to 2019 [2]. These risk factors also precipitate other conditions, such as diabetes and dyslipidaemia, that further increase the risk of complications of hypertension, including cardiac failure, stroke and renal failure.

Somaliland is an autonomous region in the northern part of Somalia that declared its independence in 1991 after the civil war in Somalia led to the downfall of the Somali Republic. However, it remains officially unrecognised to date [6]. Somaliland has since been stable, but its public service organisations, including healthcare services, remain weak. Information on the state of non-communicable diseases (NCDs) in Somaliland is scarce and little is known regarding the impact of NCDs and their evolution over time. Worthy of note, the scanty information available from the 2020 report of the Somaliland Health and Demographic Survey (SLHDS) [7] shows that hypertension (41.1%) and diabetes (19.1%) are the most prevalent chronic disease entities in adults in Somaliland. A cross-sectional study of 955 females and 145 males, aged 20–53 years, conducted in Hargeisa using household member interviews also reported high rates of behavioural and biological risk factors for NCDs in Somaliland, including hypertension (36.1% in females and 33.1% in males) [8]. In addition, high rates of obesity and overweight were reported, particularly among females [9].

Several studies revealed that hypertensive individuals with comorbidities are at increased risk for CVDs [10,11]. Interventions to prevent the complications of these comorbidities are warranted. Community-based and health facility-based studies are required to uncover the real burden of NCDs in Somaliland, so that initiatives can be tailored to specific settings.

Although household-based studies produce more representative data than health facility-based studies, the former may miss information on specific populations (e.g., hospitalised patients and patients receiving medications). The purpose of this study was to gain new insights regarding the occurrence of hypertension and related risk factors of adult patients seeking care at a major health facility in Hargeisa. We focused on the outpatient population who were seeking care from Hargeisa group hospital.

## 2. Materials and Methods

### 2.1. Study Design and Sampling

We conducted a hospital-based cross-sectional study at the main referral and University of Hargeisa teaching hospital in Hargeisa city, Somaliland, from 13 June to 15 August 2022. A total of 324 patients, aged 18 years and above, were included.

Built in 1951, Hargeisa group hospital (HGH) is the national and largest referral hospital in Somaliland. With more than 260 daily patient visits and 400 beds [12], it provides essential health services to more than a million inhabitants of Hargeisa, the capital of Somaliland. In addition, it offers referral care for other regions and districts of Somaliland.

Sample size calculation of proportions based on specified precision of estimate and desired level of confidence was applied [13]. Assuming a hypertension prevalence of 41% [7], considering a 95% confidence level, a desired precision of 5%, a none response rate of 25% and rounding the result to the next 10th digit, the required sample size to estimate the true prevalence of hypertension with the desired precision and confidence level was computed to 470.

A systematic sampling technique of participants was used, and a small pilot study preceded the actual data collection. The pilot study aimed to obtain information about the number of patients who would be enrolled per day and to consolidate data collectors’ understanding of the data collection instruments. The pilot study revealed that on average, 60 patients visited the adult outpatient department per day. A qualified nurse interviewed participants with a pretested questionnaire, while another one collected anthropometric measurements. Based on the pilot study outcome, we fixed the sampling interval to six. The first individual recruited for the study was selected randomly from the first six visitors on the first day of data collection. Using the calculated interval, we screened every sixth individual for inclusion. In case the selected individual refused or did not meet the inclusion criteria, the immediate next individual was screened for inclusion.

### 2.2. Inclusion and Exclusion Criteria

The study included individuals aged 18 years and older, regardless of their sex, who agreed to participate and provided their consent. Excluded were individuals who were unable to respond to the interviewer-administered questionnaire due to difficulties in their physical or mental well-being. Additionally, pregnant women were excluded from the study, as blood pressure distribution in pregnant women is generally different from the general population.

### 2.3. Data Sources

The study used a World Health Organisation (WHO) STEPwise Surveillance (WHO-STEPS) approach to obtain patient information. We interviewed patients face-to-face and obtained physical measurements via a modified WHO-STEPS questionnaire [14]. Our interview covered the first two steps of the WHO-STEPS questionnaire. The first step collected information about sociodemographic, behavioural and biological factors, while the second step measured physical quantities (anthropometric measurements). The physical measurements included blood pressure, body weight and height. Blood pressure was measured using a standard cuff (aneroid sphygmomanometer) after participants rested for 5 min. Standard blood pressure measurement was determined at the right arm while the participant was seated on a chair with their arm supported by a table at the heart level. Three consecutive blood pressure measures with 30 sec resting interval time in between were recorded. Participants’ blood pressure was computed as an average of the three readings. Body weight (in kg) and height (in cm) were measured using an adult weight scale with a stadiometer.

### 2.4. Definitions of Variables

Hypertension was defined as being told by a doctor or other health professional that the participant has hypertension, being on antihypertensive medications or having a measured systolic blood pressure ≥ 140 mmHg or diastolic blood pressure ≥ 90 mmHg. According to the International Society of Hypertension (ISH), blood pressure level is classified into four categories: normal (<130/85 mmHg), high-normal (130–139/85–89 mmHg), grade 1 hypertension (140–159/90–99 mmHg) and grade 2 hypertension (≥160/100 mmHg) [15].

Diabetes, in the absence of blood sugar measurement, was defined as being told by a doctor or other health professional that the participant has diabetes, or as being on hypoglycemic medications. Obesity was defined as body mass index (BMI) ≥ 30 kg/m^2^. Cholesterolaemia, in the absence of cholesterol measurement, was defined as being told by a doctor or other health professional that the participant has raised cholesterol, or as being on medications to lower cholesterol.

Khat consumption was defined as answering “yes” to the question “Have you ever chewed Khat?” Khat is the leaves and tops of the Khat tree (*Catha edulis*) chewed for their psychostimulant properties in the Horn of Africa and the southern Arabian peninsula [16]. Smoking was defined as answering “yes” to the question “Have you ever smoked any tobacco product?”

### 2.5. Data Analysis

Descriptive findings were summarised in tables and figures. The primary endpoint was the presence of hypertension. A priori selected determinants investigated for the association of hypertension were sociodemographic factors (age, sex, income and employment status), behavioural factors (smoking and Khat consumption) and biological factors (presence of diabetes, cholesterolaemia and obesity). Continuous data were presented as mean and standard deviation (SD). Categorical data were presented as frequency and relative frequency. Univariate and multivariate analyses were conducted using logistic regression to assess the relationship between hypertension and associated risk factors. Separate models were fitted for females and males. Odds ratio (OR) with 95% confidence interval (CI) was considered as the primary measure of effect sizes. We used R statistical programme version 4.1.3 [17] for the analysis.

## 3. Results

### 3.1. Study Flow and Participants’ Characteristics

**Figure 1:**
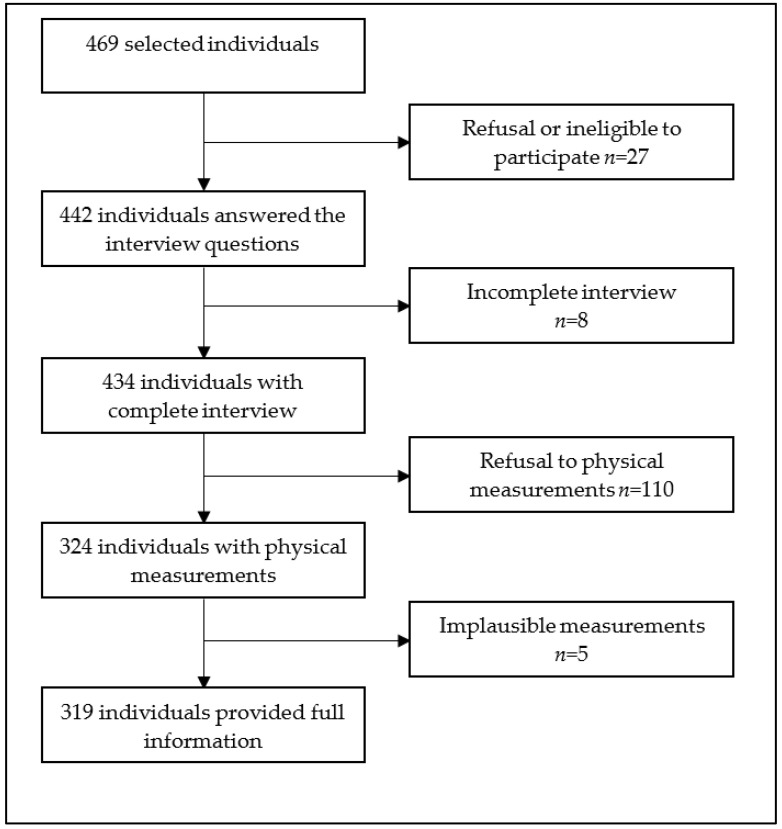
Study population flow diagram, Hargeisa, Somaliland, 2022.

Overall, 469 individuals were invited to participate. Among them, 319 (68.0%) had complete data records and were subjected to further analysis (Figure 1).

Characteristics of the study participants are summarised in Table 1. There were more males than females (173 vs. 146). Participants were aged between 20 and 80 years (mean age: 40.4 years, SD: 14.0 years). The age group 25–34 years represented more than a third of the participants (36.7%), followed by the age group 35–44 years (27.6%). The majority of participants were married (68.7%) and only attended primary school (56.7%). Employment was reported in 51.1% of participants, and among these, 64.9% earned less than the average reported monthly income.

Regarding common behavioural factors, smoking and Khat consumption were reported among 16.3% and 20.4% of participants, respectively. The behavioural factors varied considerably between males and females and across age groups. None of the females smoked or consumed Khat.

In regard to the self-reported NCDs, 30 (9.4%) and 43 (13.5%) of the participants reported having had diabetes and elevated cholesterol, respectively. Females had a significantly higher proportion of diabetes compared to males (13.7% vs. 5.8%, *p* = 0.016). Elevated levels of cholesterol were similar in both sexes (13.5% in females versus 13.3% in males). Anthropometric measurements revealed that 10 (3.1%) of the participants were obese. Obesity was more prevalent in females (5.5%) than in males (1.2%). However, neither class II type of obesity (BMI 35–39.9 kg/m^2^) nor class III type of obesity (BMI ≥ 40 kg/m^2^) were observed.

### 3.2. Prevalence of Hypertension

**Figure 2:**
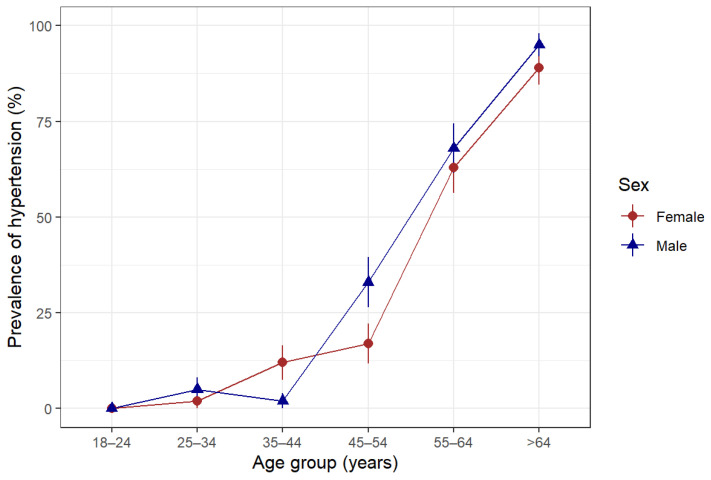
Prevalence of hypertension according to age group and sex in Somaliland, 2022. Hypertension was defined as being told by a doctor or other health professional that the patient has hypertension or being on antihypertensive medications or having a measured systolic blood pressure ≥ 140 mmHg or diastolic blood pressure ≥ 90 mmHg.

The overall prevalence of hypertension was 22.6% among the study participants. Hypertension increased with age and was higher in males than females, except for the 35-to 44-year-old age group, where females had a higher proportion of hypertension than their male counterparts (Figure 2).

According to the ISH classification of blood pressure levels, the frequency distribution of blood pressure as normal, high-normal and hypertension was 78.7%, 3.4% and 17.9%, respectively (Table 2).

**Figure 3:**
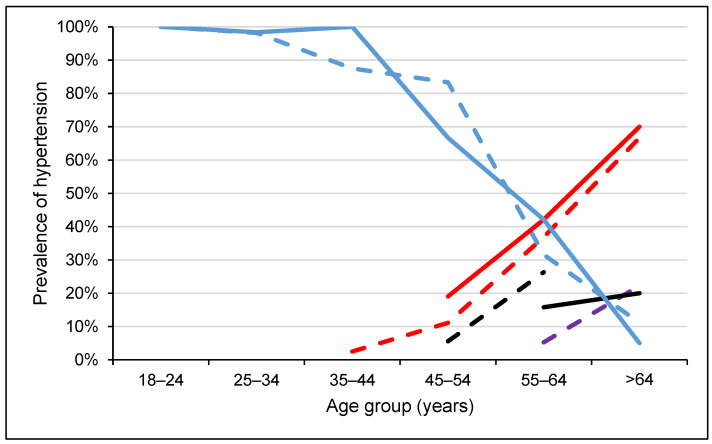
Blood pressure levels according to ISH classification across age groups, stratified by sex in Somaliland, 2022. Dash lines represent females while solid lines represent males. Red colour indicates grade 1 hypertension (SBP = 140–159 or DBP = 90–99), black colour indicates grade 2 hypertension (SBP ≥ 160 or DBP ≥ 100), violet colour indicates high normal blood pressure (SBP = 130–139 or DBP = 85–89) and blue colour indicates normal (optimal) blood pressure (SBP < 130 or DBP < 85).

Among the participants with blood pressure levels in the hypertensive range, 43 (75.4%) were grade 1 and 14 (24.6%) were grade 2. Slightly less than two-thirds of the participants with high-normal blood pressure (63.6%) were females, whereas nearly three-fifths of the participants with hypertension (59.6%) were males. On the other hand, among female participants, 4.8% and 15.8% were in the high-normal and hypertensive ranges, respectively. Among males, the corresponding proportions were 2.3% and 19.7%, respectively. The prevalence of hypertension increased with age (Figure 3). In the age groups 35–44 and 45–54 years, about two-thirds of the participants showed high-normal blood pressure, while 81.4% of the participants with grade 1 hypertension were 55 years and above. Among the age groups 35–44 and 45–54 years, only 6.3% had hypertension, while among those who were 55 years and above, the prevalence of hypertension was 70.1%.

Concerning the behavioural and biological characteristics, the prevalence of hypertension was higher among smokers, Khat consumers, obese individuals and those with co-morbidities. For instance, the proportion of participants with high-normal blood pressure who smoked was 36.4%, while the proportion of participants with grade 1 hypertension who smoked was 48.8%. Similarly, the corresponding proportions in Khat consumption were 36.4% and 53.5%, respectively. Moreover, the prevalence of hypertension was higher among individuals with diabetes than among non-diabetic individuals (36.7% versus 15.9%) and with high cholesterol than among individuals without elevated cholesterol (69.8% vs. 9.8%).

### 3.3. Risk Factors Associated with Hypertension

Unadjusted risk factor analysis from regressing the presence of hypertension on its determinants showed that hypertension was statistically significantly associated with age, educational attainment, smoking, Khat consumption, diabetes, cholesterolaemia and obesity. In particular, the univariate logistic regression model indicated that the risk of hypertension increased with age (OR = 1.17, 95% CI: 1.14–1.22) and with BMI (OR = 1.23, 95% CI: 1.12–1.35).

Our analysis also indicated that hypertension was higher in smokers (OR = 10.20, 95% CI: 5.32–20.00), in obese participants (OR = 15.30, 95% CI: 3.73–103.00), in Khat users (OR = 7.52, 95% CI: 4.14–13.90), those having diabetes (OR = 4.76, 95% CI: 2.19–10.40) and participants with high cholesterol (OR = 28.30, 95% CI: 12.70–70.00). While these associations showed statistical significance at *p* < 0.001, most of the associations were not maintained upon mutual adjustment in a multivariate logistic model. After adjustment for age, sex, educational attainment and other covariates that may affect hypertension, the risk of hypertension increased with age with an adjusted OR of 1.13 (95% CI: 1.09–1.19, *p* < 0.001), was greater in individuals with high cholesterol with an adjusted OR of 3.70 (95% CI: 1.25–11.76; *p* = 0.018) and was greater in obese participants with an adjusted OR of 17.60 (95% CI: 1.68–217.00, *p* = 0.016). None of the other covariates were significantly related to hypertension in the adjusted model (Table 3).

We conducted separate analyses for females and males. For females, the presence of hypertension was regressed against age, educational attainment, diabetes, raised cholesterol and obesity. For males, we included smoking and Khat consumption as additional covariates to the logistic model containing the above-mentioned covariates. The results indicated that in females, hypertension was significantly related to age and obesity. The odds of having hypertension increased with age with an adjusted OR of 1.16 (95% CI: 1.09–1.25, *p* < 0.001) and were higher among obese females with an adjusted OR of 44.2 (95% CI: 2.37–1784, *p* = 0.009). In males, the results showed that the odds of having hypertension increased with age with an adjusted OR of 1.12 (95% CI: 1.05–1.21, *p* < 0.001) and were higher in participants with raised cholesterol with an adjusted OR of 6.64 (95% CI: 1.21–58.8, *p* = 0.028). The data indicate that hypertension is significantly associated with age and the presence of cholesterolaemia in males (Table 4).

## 4. Discussion

We found a high prevalence of hypertension and determined its underlying risk factors in patients seeking care in health facilities in Hargeisa city, Somaliland. Indeed, almost one-quarter of the 319 participants aged 20–80 years seeking care at HGH suffered from hypertension. Age, obesity and the presence of high cholesterol levels were significantly related to hypertension. Additionally, we observed high rates of smoking and Khat consumption among males. On the other hand, high rates of diabetes, cholesterolaemia and obesity were observed in both males and females. Taken together, our findings suggest that the outpatient population in Somaliland hospitals is at a considerable burden of NCDs.

However, the prevalence of hypertension in our study (22.6%) is lower than the one stated in the SLHDS report 2020 (SLHDS 2020), which was reported at 41.1% [7]. It is also lower than the prevalence reported in an earlier household-based cross-sectional study in Hargeisa (33.1%) [8]. Nevertheless, it is comparable to results from a similar study reported in a hospital-based cross-sectional study in Mogadishu city, Somalia (21.2%) [18], a systematic review on the prevalence of hypertension reported from Ethiopia (18.1%) [19], a WHO-STEP-wise approach survey in Kenya (24.5%) [20] and a pooled analysis of global data on hypertension (24.1%) [21]. However, direct comparison among these studies comes with a notable concern due to variations in the studied populations, settings, timing and duration of the studies and methodologies.

Our study found that the prevalence of hypertension increased with age in both males and females, corroborating previous investigations [8,22,23]. Age-related increase in blood pressure is partly explained by changes in the cardiovascular system, such as stiffening of the walls of the aorta and arteries in older age groups [24], which is due to the accumulation of molecular and cellular damage of the endothelial lining [25].

Regarding the rates of smoking and Khat consumption in males, our findings are in line with Ahmed and colleagues [8], but are higher than those of the SLHDS report 2020, which reported rates of 14.3% and 18.2%, respectively [7]. Considering the state of smoking in the neighbouring countries, our findings were higher than those in Kenya (13.5%) [26,27], Sudan (19.6%) [28] and Ethiopia (21.2%) [29], but were comparable to those in Yemen (33.1%) [30]. Globally, the prevalence of smoking was 36.7% in males and 7.8% in females in 2020 [31]. Smoking is an established risk factor for hypertension and other CVDs [32,33,34,35,36]. A possible explanation of how smoking increases blood pressure is that smoking causes acute vascular injury that leads to impairment of epithelial function [37] and stimulates atherosclerosis [38].

Similarly, high rates of Khat consumption in neighbouring countries were noted: 36.8% in Kenya [39], 23.2% in Ethiopia [40] and 67.9% in Yemen [41]. Several earlier studies have presented Khat as an important risk factor for CVDs, including hypertension [42,43,44,45]. The fresh buds and leaves of the Khat plant, chewed mainly by males, contain amphetamine-like compounds (cathinone and cathine), which have vasoconstrictor activity. Hence, Khat consumption increases blood pressure and heart rate [43,46]. While these established risk factors increase blood pressure, other emerging risk factors such as air pollution also contribute to the increasing prevalence of hypertension in LMICs [47,48]. In particular, household air pollution due to the combustion of solid fuels used for cooking poses the highest risk for females. Hence, future research has to incorporate an in-depth assessment of the role of indoor and outdoor air pollutants on hypertension in females. Moreover, further analysis is warranted to investigate the association between Khat and tobacco consumption with hypertension, taking into consideration characteristics such as the age of onset, frequency and duration of consumption. This may inform future prevention and control strategies.

Consistent with prior work [49,50,51,52,53,54], our study showed that diabetes, hypercholesterolaemia and obesity are common comorbidities with hypertension. However, the rates in our study were lower than those of Ahmed and colleagues [9] and those presented in the SLHDS report 2020 [7]. For instance, 5.5% of the females in our study were obese, while the corresponding rate was 12.0% in the SLHDS report 2020 and 31.3% in Ahmed and colleagues [9]. One potential explanation for this difference is the strong independent association between relative affluence and hypertension. In Somaliland, most of the population depend on private institutions for their medical care and more than 98% of the people pay their medical expenses on their earning [7]. Only a small percentage of the population with chronic diseases utilise public institutions and most of them belong to the lower income quintiles. Hence, our study could have underestimated the actual prevalence of hypertension since our sample was drawn from patients attending a public hospital. Further analysis is warranted to comprehensively investigate the existence of metabolic syndrome and comorbidities between hypertension, diabetes, hypercholesterolaemia and obesity in outpatients attending HGH and other similar settings.

Our findings imply that nearly one-fourth of the outpatient population in Hargeisa city, Somaliland, is at increased risk for CVDs, such as heart failure, kidney failure and stroke, thus underscoring the importance of strategies to prevent and properly manage NCDs in Somaliland. We hope the Somaliland Ministry of Health Development (MoHD) will consider appropriate actions to tackle the burden and implications of chronic diseases earlier, including hypertension. Given the paucity of available evidence and lack of established clinical guidelines in Somaliland, we do not provide a decision-making algorithm for hypertension. However, we suggest the implementation of contextualised collaborative interventions to prevent and combat NCDs. Of note, the WHO Package of Essential Non-communicable disease interventions (PEN) is a set of evidence-based interventions that are designed to address the major NCDs, such as diabetes, hypertension, cancer and respiratory diseases, in primary health care settings. The package is an affordable and scalable model that is feasible in resource-limited settings such as Somaliland.

To our knowledge, this is the first hospital-based study on hypertension in Somaliland. The systematic sampling strategy applied strengthens the generalisability of our findings to other health facilities. However, our study has some limitations. First, we specifically targeted patients attending a referral hospital, which may have introduced selection bias. Second, because of the cross-sectional study design employed, we could not establish a causal relationship between the reported risk factors and hypertension. Third, the absence of biochemical measurements, due to financial constraints, limited the diagnostic assurance of self-reported conditions such as diabetes and raised cholesterol. Fourth, while chronic conditions such as hypertension are not typically subject to seasonality, a study duration of only 2 months may have limited the scope of important events. A longer-term study spanning at least one year would provide more comprehensive and representative findings. Fifth, we did not consider the duration of tobacco and Khat consumption in our risk factor analysis. However, such information is necessary to deepen the understanding of the potential impact of longer-term tobacco and Khat use on hypertension. Finally, we did not stratify our analysis into rural and urban settings as we lacked this kind of information from the study participants.

## 5. Conclusions

Our findings show that nearly a quarter of the adult outpatient population in Hargeisa city in Somaliland have hypertension. Hypertension correlates with low levels of education and obesity, and with smoking and Khat consumption in males. It follows that reducing obesity, creating public health education programmes and creating or strengthening policies and programmes against smoking and Khat consumption might have far-reaching impacts on lowering hypertension and reducing the risk of CVDs. Hence, our results and considerations underscore the importance of strategies to prevent and properly manage hypertension and other chronic NCDs in Somaliland.

## Figures and Tables

**Table 1:** Characteristics of the study participants in Somaliland, 2022 (*N* = 319).

Characteristic	Females (*n* = 146) ^1^	Males (*n* = 173) ^1^	*p*-Value
Age group (years)			0.585
18–24	5 (3.4%)	3 (1.7%)	
25–34	55 (37.7%)	62 (35.8%)	
35–44	40 (27.4%)	48 (27.7%)	
45–54	18 (12.3%)	21 (12.1%)	
55–64	19 (13.0%)	19 (11.0%)	
≥65	9 (6.2%)	20 (11.6%)	
Marital status			0.001
Single	29 (19.9%)	50 (28.9%)	
Married	100 (68.5%)	119 (68.8%)	
Divorced	17 (11.6%)	4 (2.3%)	
Educational attainment			0.003
Primary school or less	99 (67.8%)	82 (47.4%)	
Secondary school	19 (13.0%)	32 (18.5%)	
University/college	28 (19.2%)	59 (34.1%)	
Employment status			<0.001
Employed	48 (32.9%)	115 (66.5%)	
Unemployed	97 (66.4%)	57 (32.9%)	
Income quantile			<0.001
Low	54 (37.0%)	32 (18.5%)	
Lower middle	51 (34.9%)	60 (34.7%)	
Upper middle	32 (21.9%)	59 (34.1%)	
High	3 (2.1%)	18 (10.4%)	
Unknown	6 (4.1%)	4 (2.3%)	
Smoking	0 (0.0%)	52 (30.1%)	<0.001
Khat consumption	0	65 (37.6%)	<0.001
Diabetes	20 (13.7%)	10 (5.8%)	0.016
Cholesterolaemia	20 (13.7%)	23 (13.3%)	0.916
Hypertension	29 (19.9%)	43 (24.9%)	0.288
Systolic blood pressure	121.3 ± 17.3	123.0 ± 16.3	0.192
Diastolic blood pressure	77.8 ± 14.3	79.6 ± 13.1	0.062
Height (in cm)	165.5 ± 6.8	171.2 ± 6.3	<0.001
Weight (in kg)	68.7 ± 8.2	66.9 ± 6.5	0.067
Body mass index (BMI)			<0.001
Underweight	0	2 (1.2%)	
Optimal weight	70 (47.9%)	140 (80.9%)	
Overweight	68 (46.6%)	29 (16.8%)	
Obese	8 (5.5%)	2 (1.2%)	

^1^ mean ± SD for continuous variables; *n* (%) for categorical variables.

**Table 2:** Prevalence of hypertension according to ISH classification in Somaliland, 2022 (*N* = 319).

Characteristic	Normal (*n* = 251)	High-Normal (*n* = 11)	Grade 1 Hypertension (*n* = 43)	Grade 2 Hypertension (*n* = 14)
Sex				
Female	116 (46.2%)	7 (63.6%)	16 (37.2%)	7 (50.0%)
Male	135 (53.8%)	4 (36.4%)	27 (62.8%)	7 (50.0%)
Age group (years)				
18–24	8 (3.2%)	0 (0.0%)	0 (0.0%)	0 (0.0%)
25–34	115 (45.8%)	0 (0.0%)	1 (2.3%)	1 (7.1%)
35–44	83 (33.1%)	4 (36.4%)	1 (2.3%)	0 (0.0%)
45–54	29 (11.6%)	3 (27.3%)	6 (14.0%)	1 (7.1%)
55–64	14 (5.6%)	1 (9.1%)	15 (34.9%)	8 (57.1%)
≥65	2 (0.8%)	3 (27.3%)	20 (46.5%)	4 (28.6%)
Marital status				
Single	77 (30.7%)	0 (0.0%)	1 (2.3%)	1 (7.1%)
Married	155 (61.8%)	10 (90.9%)	41 (95.3%)	13 (92.9%)
Divorced	19 (7.6%)	1 (9.1%)	1 (2.3%)	0 (0.0%)
Educational attainment				
Primary school or less	118 (47.0%)	10 (90.9%)	41 (95.3%)	12 (85.7%)
Secondary school	49 (29.5%)	0 (0.0%)	2 (4.7%)	0 (0.0%)
University/college	84 (33.5%)	1 (9.1%)	0 (0.0%)	2 (14.3%)
Employment status				
Employed	137 (54.6%)	5 (45.5%)	13 (30.2%)	8 (57.1%)
Unemployed	113 (45.0%)	6 (54.5%)	30 (69.8%)	5 (35.7%)
Smoking	21 (8.4%)	4 (36.4%)	21 (48.8%)	6 (42.9%)
Khat consumption	31 (12.4%)	4 (36.4%)	23 (53.5%)	7 (50.0%)
Diabetes	15 (6.0%)	4 (36.4%)	11 (25.6%)	0 (0.0%)
Cholesterolemia	7 (2.8%)	6 (54.5%)	25 (58.1%)	5 (35.7%)
Body mass index				
Underweight	2 (0.8%)	0 (0.0%)	0 (0.0%)	0 (0.0%)
Optimal weight	176 (70.1%)	4 (36.4%)	23 (53.5%)	7 (50.0%)
Overweight	71 (28.3%)	6 (54.5%)	15 (34.9%)	5 (35.7%)
Obese	2 (0.8%)	1 (9.1%)	5 (11.6%)	2 (14.3%)

**Table 3:** Univariate and multivariate analysis of hypertension in Somaliland, 2022.

	Unadjusted Model			Adjusted Model		
Characteristic	OR	95% CI	*p*-Value	OR	95% CI	*p*-Value
Sex (Male)	1.33	0.79–2.29	0.289	1.07	0.27–3.83	0.917
Age	1.17	1.14–1.22	<0.001	1.13	1.09–1.19	<0.001
Educational attainment						0.418
Primary school or less	1.00	—		1.00	—	
Secondary school	0.15	0.04–0.39	<0.001	1.04	0.22–4.24	
University/college	0.06	0.02–0.18	<0.001	0.40	0.07–1.65	
Smoking	10.2	5.32–20.00	<0.001	2.51	0.53–13.00	0.265
Khat consumption	7.52	4.14–13.90	<0.001	0.81	0.13–4.46	0.827
Diabetes	4.76	2.19–10.40	<0.001	1.08	0.36–3.17	0.861
Raised cholesterol	28.3	12.70–70.00	<0.001	3.70	1.25–11.70	0.018
Obesity	15.3	3.73–103.00	<0.001	17.6	1.68–217.00	0.016

OR = odds ratio, CI = confidence interval.

**Table 4:** Risk factors of hypertension stratified by sex in Somaliland, 2022.

	Females			Males		
Characteristic	OR	95% CI	*p*-Value	OR	95% CI	*p*-Value
Age	1.16	1.09–1.25	<0.001	1.12	1.05–1.21	<0.001
Educational attainment			0.775			0.263
Primary school or less	1.00	—		1.00	—	
Secondary school	2.80	0.20–25.7		0.70	0.09–3.99	
University/college	1.40	0.07–11.8		0.19	0.02–1.17	
Smoking				2.07	0.39–11.90	0.473
Khat consumption				0.71	0.08–4.42	0.742
Diabetes	0.72	0.13–3.21	0.691	1.70	0.34–10.10	0.467
Raised cholesterol	3.17	0.63–16.2	0.210	6.64	1.21–58.8	0.028
Obesity	44.2	2.37–1784	0.009	1.17	0.00–367.0	0.945

## Data Availability

The data are stored in the Swiss Tropical and Public Health Institute repository. Data stored in this repository are shared according to the terms and conditions of the repository.
